# Modeling and prediction of 316 L stainless steel relative density in L-PBF process using machine learning

**DOI:** 10.1038/s41598-026-58664-y

**Published:** 2026-06-23

**Authors:** Saleh Asnaashari, Sadegh Yousefi, Maria P. Nikolova

**Affiliations:** 1 School of Metallurgy and Materials Engineering, University College of Engineering, University of Tehran, Tehran, Iran; 2 Ferdows Department of Education (Ministry of Education), Experimental Sciences Teacher, Ferdows, Iran; 3 Department of Material Science and Technology, University of Ruse “Angel Kanchev”, 8 Studentska Str, 7017 Ruse, Bulgaria

**Keywords:** Laser powder bed fusion, Relative density, K-nearest neighbours, Multilayer perceptron, AdaBoost-DT, Committee machine intelligent system, Engineering, Materials science, Mathematics and computing

## Abstract

This study investigates the prediction of relative density in L‑PBF parts using machine learning models trained on a literature-based dataset comprising 287 experimental measurements collected from previously published studies. The input parameters for the proposed models included laser power, scanning speed, and hatch spacing. To model the nonlinear relationship between process variables and relative density, k-nearest neighbours (KNN), adaptive boosting decision trees (AdaBoost-DT), and four multilayer perceptron (MLP) models optimised using Scaled Conjugate Gradient (SCG), Levenberg–Marquardt (LM), Bayesian Regularisation (BR), and Resilient Backpropagation (RB) were developed. The predictions from these six models were subsequently integrated into a single framework using a committee machine intelligence system (CMIS), which provided improved predictive performance. Model accuracy was evaluated using statistical metrics including the coefficient of determination (R²), standard deviation (SD), average percent relative error (APRE), average absolute percent relative error (AAPRE), and root mean square error (RMSE). The results showed that the proposed hybrid modelling framework can effectively capture the complex relationship between L‑PBF process parameters and relative density. However, the heterogeneity of the literature-based dataset and the presence of a limited number of outliers may introduce uncertainty into the predictions.

## Introduction

Additive manufacturing (AM) is a layer‑by‑layer fabrication technology that enables the production of complex three‑dimensional components directly from digital models with improved material efficiency and reduced manufacturing constraints^1–3^. Due to these advantages, AM has gained increasing attention in various industries such as aerospace, biomedical, and energy sectors^4^. Among different AM techniques, laser powder bed fusion (L‑PBF), also known as selective laser melting (SLM), is one of the most widely used processes for manufacturing intricate metallic components with minimal material waste^5–8^. In the L‑PBF process, metal powder layers are selectively melted by a laser heat source to form a melt pool that rapidly solidifies upon cooling. The process is repeated layer by layer until the final part is produced^9–11^.

Although seemingly straightforward, L-PBF involves complex physical and hydrodynamic phenomena, including rapid melting, solidification, vaporisation, and intricate melt-pool dynamics, which are not yet fully understood^11,12^. These phenomena are highly sensitive to process parameters such as laser power, scan speed, hatch spacing, layer thickness, and powder properties^13^. Producing high-quality parts, therefore, requires operation within a narrowly defined “process window” that balances these interacting effects^14,15^. However, identifying this window is both time- and resource-intensive, as it depends on the specific material–machine combination. This challenge constitutes a major barrier to industrial adoption and can compromise the anticipated efficiency gains of additive manufacturing^16,17^.

The relative density of parts fabricated via laser powder bed fusion (L‑PBF) is strongly influenced by key process parameters, particularly laser power, scanning speed, and hatch spacing. These parameters govern the energy input, melt pool behavior, and overlap between adjacent scan tracks, thereby affecting the formation of defects such as lack‑of‑fusion pores and keyhole porosity. Therefore, achieving high relative density requires careful optimization of the processing window^18–20^.

Achieving high-quality parts in L-PBF critically depends on the optimisation of process parameters, which has traditionally been pursued through extensive and costly trial-and-error experimentation^21^. Machine learning (ML) has emerged as a powerful means of overcoming this limitation by providing data-driven models capable of accurately predicting outcomes in complex engineering systems such as AM ^22–24^. These intelligent techniques have been successfully applied in metallurgy and materials science to assess a wide range of material properties with high accuracy^25^. Recently, machine learning (ML) approaches have been increasingly used to model complex relationships between process parameters and part quality in additive manufacturing. For example, Hassanin et al.^26^ demonstrated that a deep learning neural network outperformed conventional ML and statistical approaches in predicting the mechanical properties of titanium lattice structures. In the context of L-PBF, several studies have demonstrated the potential of ML for predicting relative density and related quality metrics. For instance, Zhang et al. ^27^(2022) used ML models with physics-based inputs such as volumetric energy density to improve density prediction, showing that domain-informed features can enhance model performance. Likewise, Scime and Beuth^28^ and Gobert et al. ^29^ applied ML-based classifiers for defect detection in L-PBF, achieving high diagnostic accuracy using human-labelled image datasets. These studies indicate that ML is capable of capturing the nonlinear process–structure–property relationships governing densification in L-PBF, while also suggesting that predictive performance can be improved when physically interpretable features are considered alongside process parameters. Collectively, these findings highlight the transformative potential of ML for process optimisation and quality control in AM. Finally, the relative density of a structure plays a critical role in determining its stiffness and strength, as well as influencing macroscopic yield criterion assumptions^30^.

Although a wide range of machine learning techniques has been applied to problems in additive manufacturing, most existing studies rely on single predictive models and focus primarily on algorithm selection, with limited attention to prediction robustness and reliability under heterogeneous conditions. In contrast, the present study introduces a Committee Machine Intelligent System (CMIS) for predicting the relative density of 316 L stainless steel fabricated via L‑PBF. By integrating heterogeneous learners—including KNN, AdaBoost-DT, and multiple neural-network models—and combining them through a data-driven weighting strategy, the proposed framework is designed to improve stability, generalisation, and prediction reliability. The novelty of this work lies not only in the ensemble formulation itself, but also in its focus on robust density prediction from a heterogeneous literature-derived dataset and its potential as a practical decision-support tool for reducing costly trial-and-error in process optimisation.

## Model development

### Data collection

The compiled dataset originates from several independent experimental studies conducted using different L‑PBF systems and processing environments, all employing 316 L stainless steel powder. While combining multiple literature sources allows the creation of a relatively large dataset, it may also introduce heterogeneity due to variations in machine configurations, powder characteristics, and processing conditions. This limitation should be considered when interpreting the predictive performance of the developed models.

The use of a comprehensive database is essential for evaluating the effectiveness of a modelling study, as models trained on extensive datasets can achieve high predictive accuracy. In this work, a large database comprising 287 experimental relative density measurements was compiled from the open literature^31–38^. The collected data were extracted only from studies that reported the same target variable (relative density) and the same key process parameters, namely laser power, scanning speed, and hatch spacing. To ensure compatibility among different literature sources, the data were carefully checked for consistency in measurement units and variable definitions before being included in the final database. Although minor variations may exist among the original experimental setups, the selected datasets fall within comparable processing ranges, which allows their integration into a unified dataset for machine learning modelling.

### Data preprocessing

Before model development, the compiled dataset was statistically analysed to better understand its characteristics. The analysis revealed that the data distribution is asymmetric and does not follow a normal distribution. The dataset was randomly divided into a training set (229 samples) and a testing set (58 samples). The training dataset was used to construct the machine learning models, while the testing dataset was employed to evaluate their predictive capability on unseen data. This strategy provides an objective assessment of model generalisation performance and reduces sampling bias.

A statistical summary of the database is provided in Table 1. The analysis reveals that the data distribution is asymmetric and does not follow a normal distribution. Skewness, which quantifies the degree of asymmetry of a distribution about its mean, equals zero for a normal distribution. A positive skewness indicates that data are concentrated toward lower values, with a longer tail toward higher values, whereas a negative skewness reflects clustering toward higher values. The shape of the distribution relative to a normal distribution can be further characterised using the kurtosis parameter. For symmetric unimodal distributions, positive kurtosis corresponds to a sharper peak and heavier tails than a normal distribution, while negative kurtosis indicates a flatter distribution with lighter tails.

**Fig. 1:**
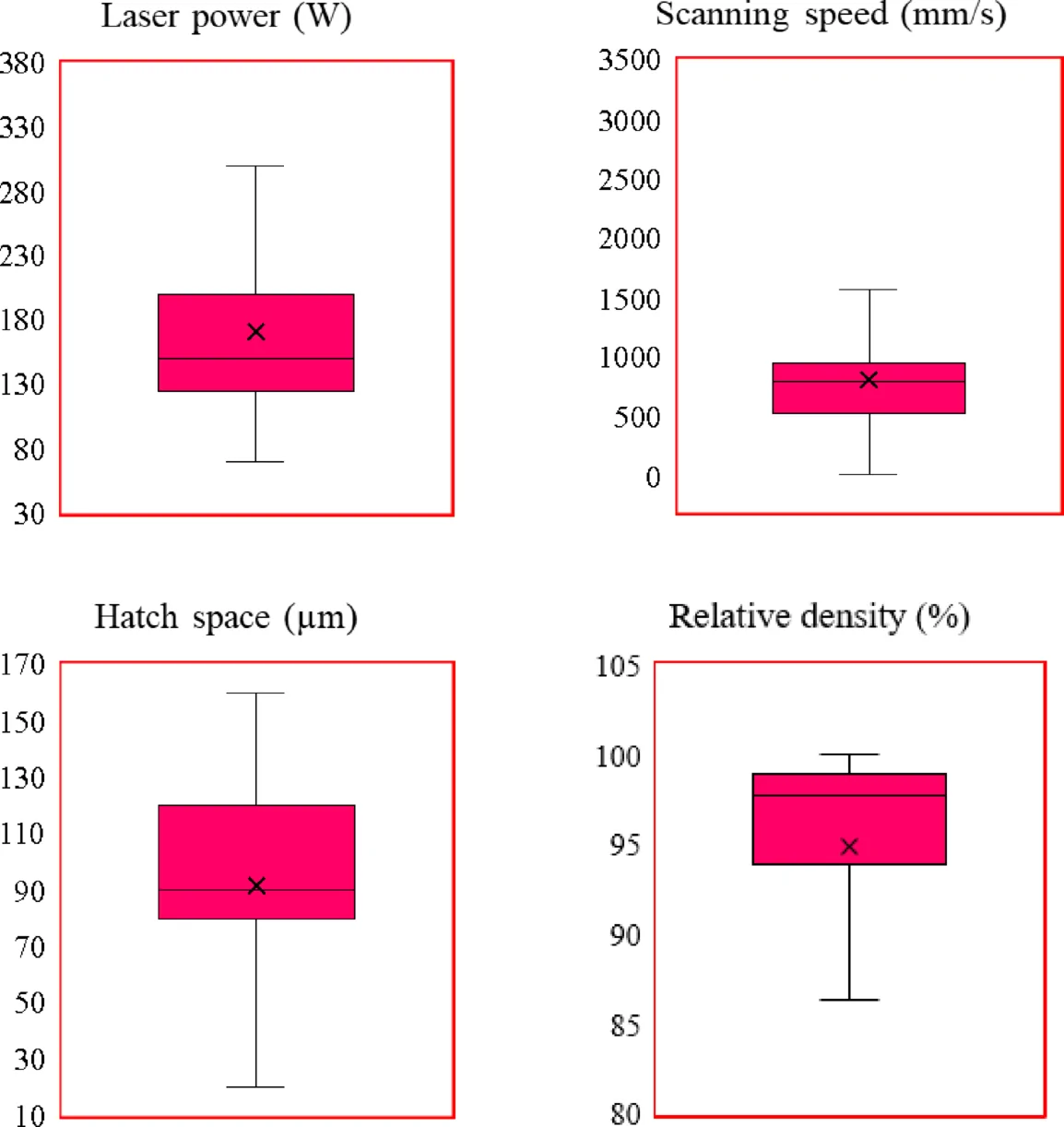
Box-and-whisker plots of the input and output data points.

A box-and-whisker plot provides an effective graphical summary of a dataset using five key statistics: the minimum and maximum values, the median, and the first and third quartiles. In such plots, the lower and upper extremes represent the minimum and maximum values, respectively, the median is shown as a horizontal line within the box, and the box spans from the first to the third quartile. Figure 1 illustrates the distributions of the input variables and target relative density values using box-and-whisker plots.

**Table 1:** Statistical characteristics of the dataset compiled in this study.

Parameter	Minimum	Maximum	Average	Mode	Skewness	Kurtosis
Laser power (W)	70.00	360.00	171.55	150.00	1.19	1.22
Scanning speed (mm/s)	18.76	3400.00	813.92	800.00	1.75	5.02
Hatch space (µm)	20.00	160.00	91.60	80.00	−0.20	−0.20
Experimental results of relative density (%)	66.18	99.99	94.90	98.25	−1.95	3.47

**Fig. 2:**
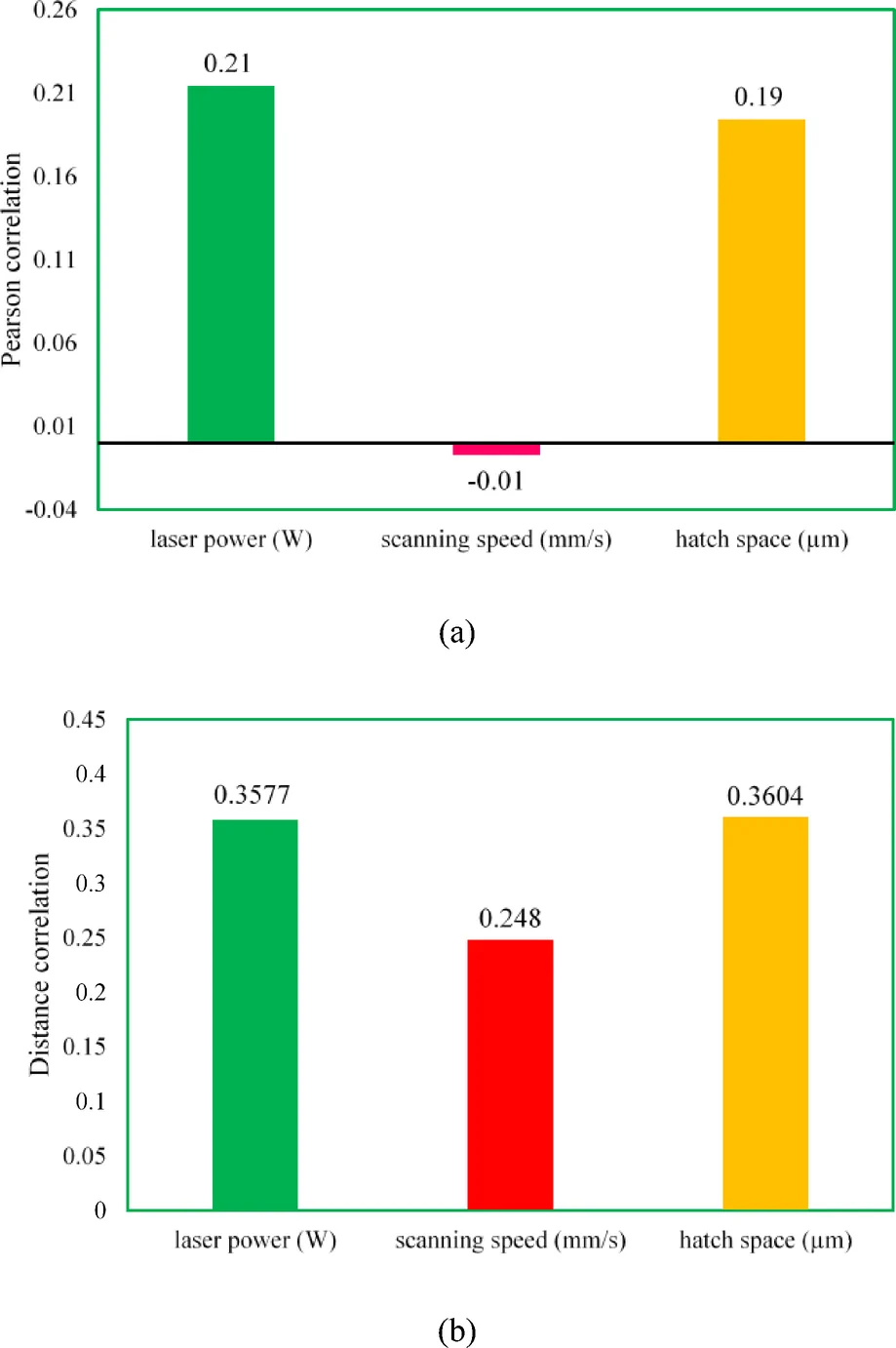
Influence of input parameters on the output, as determined by (**a**) Pearson correlation and (**b**) distance correlation.

Pearson correlation and distance correlation analyses were employed to assess both linear and linear/nonlinear relationships between the input and output variables. The Pearson correlation coefficient ranges from − 1 to 1, where negative values indicate a decrease in the output with increasing input, and positive values indicate an increase in the output as the input rises. In contrast, the distance correlation coefficient is a non-negative metric bounded within the open interval (0, 1) and is capable of capturing both linear and nonlinear dependencies. Figure 2 presents the influence of the input parameters on the output as quantified by both Pearson and distance correlation measures.

These results indicate that laser power and hatch spacing exert the strongest influence on relative density. According to Simchi’s study on the effect of laser energy on the densification of iron-based powders, increasing laser energy density raises the melt temperature, facilitating liquid flow into pores and interparticle spaces. This enhanced flow is attributed to the relatively low viscosity of the molten material, leading to increased component density^39^. However, extremely narrow scan spacings result in excessively high energy input, promoting the formation of large and irregular pores. Conversely, overly wide scan spacings produce insufficient overlap between adjacent tracks, leaving unmelted regions that give rise to aligned and excessive porosity^40^.

### K-nearest neighbors (KNN)

The k-nearest neighbour (KNN) is a non-parametric supervised learning algorithm used for both regression and classification, originally established in the early 1970s^41^. The model identifies the closest samples to a given instance where is a fixed hyperparameter and calculates proximity typically via Euclidean distance. In regression tasks, the unknown sample’s value is estimated by averaging its neighbours’ responses. Because predictive performance is highly sensitive to *k*, it is usually optimized by evaluating model accuracy across a range of candidate values^42^.

### Multilayer perceptron (MLP)

Artificial neural networks (ANNs) are computational intelligence models inspired by the human nervous system, capable of capturing complex nonlinear relationships between inputs and outputs^43^. They comprise interconnected processing units (neurons) linked by weighted connections and organised into layers, with the weights controlling connection strengths^44^. A widely used architecture is the multilayer perceptron (MLP), which includes an input layer, one or more hidden layers, and an output layer^25,45^. The input layer receives the predictors, the output layer produces the model response, and the hidden layers act as feature extractors mapping inputs to outputs^46^. In an MLP, the number of input neurons equals the number of input variables, and the output layer typically contains a single neuron representing the target property. The number of hidden layers and neurons is usually selected empirically; a single hidden layer often suffices, although more complex problems may require additional layers^47,48^. Neurons in successive layers are fully connected: each neuron computes a weighted sum of the previous layer outputs plus a bias term, which is then transformed by an activation function^49^. Different activation functions including logsig, linear, and tansig may be used in the hidden and output layers^50,51^. In this study, the most effective configuration was an MLP with two hidden layers: the first using the tansig activation function, the second using logsig, and the output layer employing a linear (purelin) transfer function. Accordingly, the model output can be expressed by considering this network structure as follows^52^:

1 $${\mathrm{Output}} = {\mathrm{purelin}}(w_{3} \times (logsig(w_{2} \times (tansig(x) + b_{1} )) + b_{2} ) + b_{3} )$$

where* w*_1_ and* w*_2_ denote the weight matrices of the first and second hidden layers,* w*_3_ corresponds to the output layer, and *b*_1_ and *b*_2_, and *b*_3_ are the associated bias terms. Four training algorithms were investigated—Bayesian Regularisation (BR), Scaled Conjugate Gradient (SCG), Resilient Backpropagation (RB), and Levenberg–Marquardt (LM)—yielding BR-MLP, SCG-MLP, RB-MLP, and LM-MLP models, respectively.

**Fig. 3:**
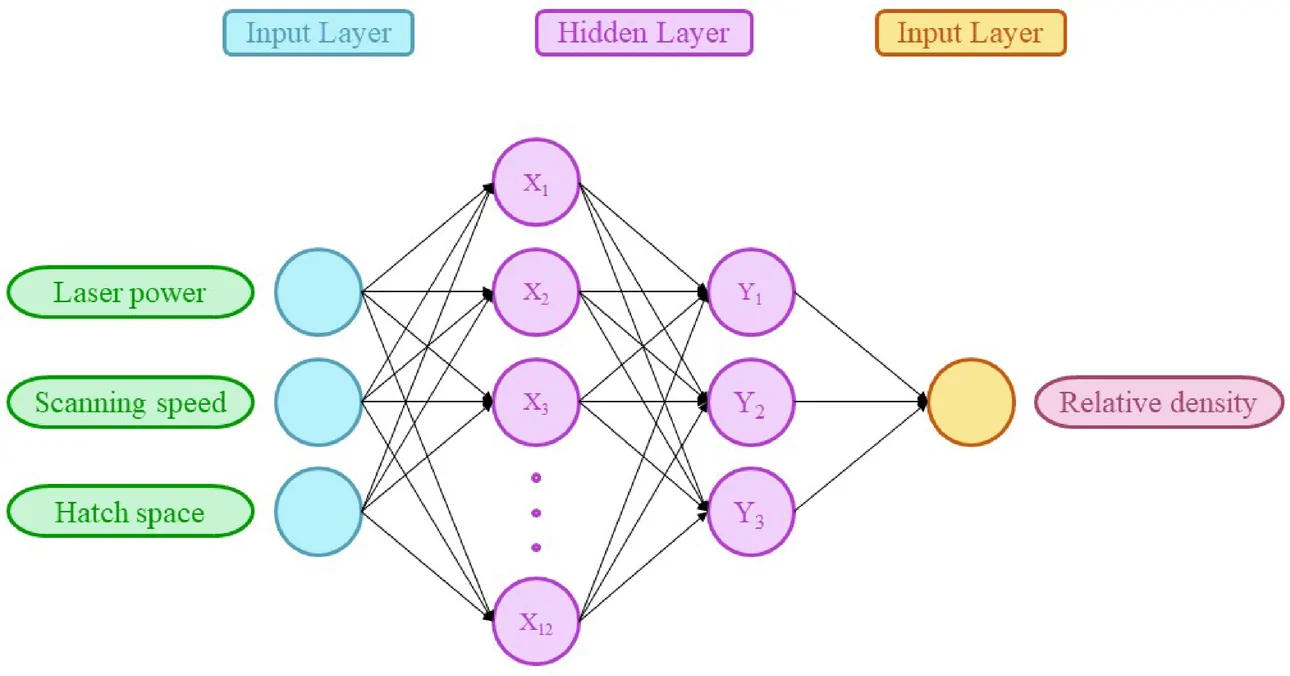
Schematic illustration of the MLP network performed in this work.

The implemented MLP architecture (Fig. 3) converged to an optimal 3–12–3–1 topology with three input neurons, twelve neurons in the first hidden layer, three neurons in the second hidden layer, and one output neuron. For each configuration, weights and biases were adjusted to minimise prediction error in relative density estimation. The 287 experimental data points were randomly partitioned into training (≈ 80%) and testing (≈ 20%) subsets to evaluate model performance and reduce sampling bias.

#### Resilient Backpropagation algorithm

The *sigmoid* and *tansig* functions, commonly used in MLP hidden layers, are “squashing” transfer functions that map an infinite input range to a finite output range. When large inputs are propagated through these functions, the resulting gradients become very small—a phenomenon known as the vanishing gradient problem, which hinders training of gradient-based methods by causing negligible weight updates. To address this issue, the Resilient Backpropagation (RB) algorithm was developed^52^.

#### Levenberg-Marquardt algorithm

The Levenberg–Marquardt (LM) algorithm is a widely used method for optimising the weights and biases in multilayer perceptron (MLP) networks. Originally developed as a damped least-squares (DLS) technique for nonlinear least-squares problems, it has been extensively validated in function approximation and is regarded as a reliable nonlinear optimisation method^53–56^. Although LM may converge to a local rather than a global minimum, it can still approach near-optimal solutions even when initialised from an inaccurate starting point. A key advantage of LM is that it avoids explicit computation of the Hessian matrix by approximating the gradient and Hessian under the assumption that the performance function is a sum of squared errors.

#### Bayesian Regularisation algorithm

The Bayesian Regularisation (BR) algorithm is employed to determine optimal network weights and biases by minimising a objective function that combines squared errors with network weights, thereby enhancing generalisation performance^57^. The objective function is defined as:

2 $$F(\omega ) = \alpha E_{\omega } + \beta E_{D}$$

where *E*_*ω*_, *E*_*D*_, and *F(ω)* represent the sum of squared model weights, the total model error, and the objective function, respectively. The coefficients $\:\alpha\:\:$and $\:\beta\:\:$in the objective function are determined using Bayes’ theorem. In this approach, weights are treated as random variables, assuming both the training set and weight vectors follow Gaussian distributions. The LM algorithm iteratively updates the weight space and re-estimates *α* and *β* until the specified stopping criteria are met.

#### Scaled conjugate gradient algorithm

In gradient-based training, weights may be updated along the steepest descent direction, corresponding to the negative gradient that provides the greatest immediate reduction in the performance function. However, this approach does not always yield the fastest convergence. The conjugate gradient method improves convergence by incorporating information from previous search directions, thereby reducing redundant movements in weight space.

3 $$p_{0} = - g_{0}$$

In Eq. (3), *−g*_*0*_ denotes the initial steepest descent direction, while *P*_*0*_ represents the conjugate search direction. The optimal step length along the current search direction is determined as shown in Eq. (4), and the new search direction is obtained by combining the current gradient with the previous direction^58^, as expressed in Eq. (5). The parameter$\:\:\beta\:$ controls the degree of conjugacy in the gradient direction. To further improve computational efficiency, the Scaled Conjugate Gradient (SCG) algorithm can be used as an alternative to the traditional line search procedure.

4 $$x_{{k + 1}} = x_{k} + \alpha _{k} g_{k}$$

5 $$P_{k} = - g_{k} + \beta _{k} P_{{k - 1}}$$

### Adaptive boosting decision tree (AdaBoost-DT)

The AdaBoost-DT algorithm integrates the strengths of boosting and decision trees to construct a more effective predictive model. The boosting technique, introduced by Schapire^59^, involves the iterative training of multiple weak learners, with each successive learner focusing on correcting the errors of its predecessors. This sequential approach produces a strong and robust ensemble model that typically outperforms the performance of the individual weak learners.

#### Adaptive boosting (AdaBoost)

AdaBoost is a widely used boosting algorithm introduced by Freund and Schapire in 1995 for classification tasks^60^. The method begins by assigning equal weights to all training samples. In each subsequent iteration, the weights of misclassified samples are increased, forcing the new learner to focus on the more challenging instances.

#### Decision tree (DT)

This non-parametric supervised learning method is applicable to both regression and classification tasks. The Automatic Interaction Detection (AID) decision tree, developed by Morgan and Sonquist, was later extended by the THAID method—the first classification tree model introduced by Messenger and Mandell^61^. A decision tree is a hierarchical structure consisting of a root node, internal (test) nodes, branches, and leaf nodes. The root node represents the entire dataset, internal nodes define decision splits, and leaf nodes correspond to the final outcomes.

**Fig. 4:**
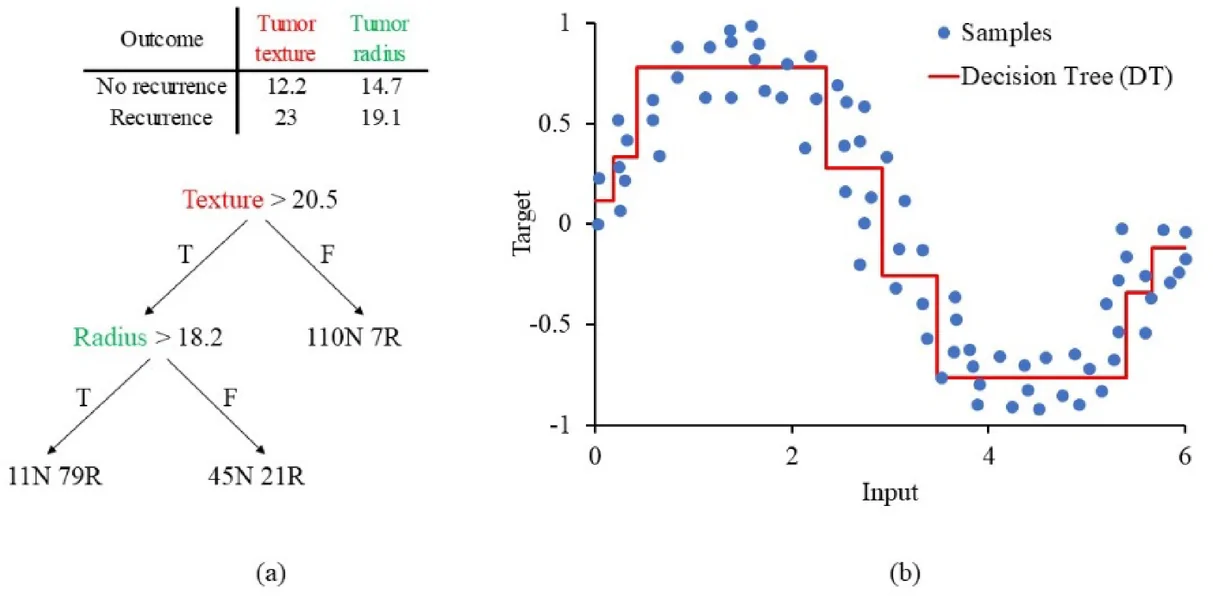
Decision tree-based examples of (**a**) classification and (**b**) regression problems.

Decision tree construction involves three main stages: splitting, stopping, and pruning^62^. Splitting divides the data into subsets based on the most informative attributes, using criteria such as standard deviation reduction, variance reduction, classification error, Gini index, gain ratio, information gain, or towing^63^. As illustrated in Fig. 4, the data are recursively partitioned from the root node to internal nodes until predefined stopping or homogeneity conditions are satisfied.

Stopping criteria are essential for controlling tree complexity. These criteria can include the minimum number of records required in a leaf or node before a split is allowed, or the distance from the leaf to the root node. Applying these constraints prevents overfitting: without them, the tree could grow excessively, producing nodes that are 100% pure for the training data but generalise poorly to unseen test data. If stopping criteria alone are insufficient, pruning can be employed to further reduce overfitting. In pruning, a full tree is first constructed, after which nodes that contribute little to validation performance or information gain are removed, resulting in a more compact and generalizable tree.

To obtain optimal predictive performance, hyperparameter tuning was carried out for each machine learning model. A guided trial-and-error approach was adopted, in which different combinations of model parameters were systematically tested using validation data. The optimal hyperparameters were selected based on their ability to minimise statistical error metrics while maintaining stable and reliable predictive performance.The optimal values of the key hyperparameters for the machine learning models employed in this study are presented in Table 2.

**Table 2:** Optimal hyperparameter values for the proposed models.

	Parameters	Value
AdaBoost-DT	Loss function	Exponential loss
	n_estimators	100
	max_depth	15
	min_samples_split	3
	min_samples_leaf	1
	max_features	All
MLP	Training function	SCG, BR, LM, RB
	Number of layers	3
	Neurons in the 1st hidden layer	12
	Transfer function of 1st hidden layer	Tansig
	Neurons in the 2nd hidden layer	3
	Transfer function of the 2nd hidden layer	Logsig
	Transfer function of the output layer	Purelin
KNN	n_neighbors	2

### Committee machine intelligent system (CMIS)

In conventional approaches to developing intelligent models, researchers often generate multiple candidate models and select the best-performing one, discarding the others. This practice can result in wasted effort on training models that are ultimately not used. To address this limitation, Nilsson introduced the Committee Machine Intelligent System (CMIS) in 1965 ^64^. CMIS is a type of artificial neural network that combines multiple models to solve a problem, employing a “divide and conquer” strategy.

CMIS models are generally classified into two categories: static structure and dynamic structure^65^. The method adopted in this study corresponds to a static structure model, where the key idea is to integrate the outputs of several expert models to produce a more accurate and reliable overall prediction.

One approach for integrating the outputs of multiple models is through linear combinations, such as simple or weighted averaging^66,67^. This strategy allows the expertise of individual models to be combined, yielding a more robust and accurate final prediction. However, simple averaging has a limitation: it assigns equal importance to all models, regardless of their individual accuracy, whereas more reliable solutions should have a greater influence on the overall result.

In the proposed method, the contributions of individual models are enhanced using a modified weighted averaging technique, where the weights are determined based on the accuracy of each model. The sum of the weights in the linear combination is constrained to equal one^68^. Accordingly, the contribution of each model to the CMIS output is proportional to the absolute value of its corresponding coefficient in the linear equation, ensuring that more accurate models exert a stronger influence on the final prediction compared to traditional weighted averaging approaches.

## Results and discussion

### Evaluation of the models statistically

The predictive performance of the developed models was evaluated using several statistical indicators, including the coefficient of determination (R^2^), standard deviation (SD), average percent relative error (APRE), average absolute percent relative error (AAPRE), and root mean square error (RMSE). Table 3 presents these parameters for the proposed models, including the coefficient of determination (R^2^), standard deviation (SD), average absolute per cent relative error (AAPRE), root mean square error (RMSE), and average per cent relative error (APRE).

R^2^:

6 $$R^{2} = \frac{{\left( {\sum\limits_{{i = 1}}^{N} {\left( {RD_{{exp,i}} - \overline{{RD}} _{{exp}} } \right)\left( {RD_{{pred,i}} - \overline{{RD}} _{{pred}} } \right)} } \right)^{2} }}{{\sum\limits_{{i = 1}}^{N} {\left( {RD_{{exp,i}} - \overline{{RD}} _{{exp}} } \right)^{2} \sum\limits_{{i = 1}}^{N} {\left( {RD_{{pred,i}} - \overline{{RD}} _{{pred}} } \right)^{2} } } }}$$

1. SD:

7 $$SD = \left( {\frac{1}{{N - 1}}\sum\limits_{{i = 1}}^{N} {\left( {\frac{{RD_{{exp,i}} - RD_{{pred,i}} }}{{RD_{{exp,i}} }}} \right)^{2} } } \right)^{{\frac{1}{2}}}$$

2. AAPRE:

8 $${\mathrm{AAPRE}} = \frac{{100}}{N}\sum\limits_{{i = 1}}^{N} {\left| {\frac{{RD_{{exp,i}} - RD_{{pred,i}} }}{{RD_{{exp,i}} }}} \right|}$$

3. RMSE:

9 $${\mathrm{RMSE}} = \left( {\frac{{\sum\limits_{{i = 1}}^{N} {\left( {RD_{{exp,i}} - RD_{{pred,i}} } \right)^{2} } }}{N}} \right)^{{\frac{1}{2}}}$$

4. APRE:

10 $${\mathrm{APRE}} = \frac{{100}}{N}\sum\limits_{{i = 1}}^{N} {\left( {\frac{{RD_{{exp,i}} - RD_{{pred,i}} }}{{RD_{{exp,i}} }}} \right)}$$

In the equations presented, *RD* denotes the relative density, $\:\stackrel{-}{RD}$, represents the mean of the relative density values, and *N* is the size of the dataset. The superscripts *exp* and *pred* refer to the experimental and predicted values, respectively, as reported by the models used in this study.

**Table 3:** Statistical evaluation of the models developed for predicting the relative density of 316 L stainless steel.

	AdaBoost-DT	SCG-MLP	LM-MLP	BR-MLP	RB-MLP	KNN	CMIS
*Training set*							
R^2^	0.676	0.780	0.777	0.759	0.785	0.688	0.800
SD	0.049	0.037	0.038	0.037	0.037	0.049	0.036
APRE (%)	0.291	−0.127	−0.138	−0.147	−0.132	1.206	−0.107
AAPRE (%)	1.949	2.007	1.927	2.022	2.078	2.041	1.836
RMSE	4.245	3.052	3.171	3.181	3.132	4.332	2.979
Num. of data	229	229	229	229	229	229	229
*Testing set*							
R^2^	0.733	0.690	0.779	0.715	0.624	0.480	0.815
SD	0.041	0.040	0.029	0.041	0.037	0.046	0.029
APRE (%)	−0.141	0.168	0.110	−0.053	0.308	0.301	0.679
AAPRE (%)	2.044	2.274	1.745	2.718	2.367	2.481	1.575
RMSE	3.559	3.624	2.578	3.570	3.363	3.968	2.692
Num. of data	58	58	58	58	58	58	58
*Total*							
R^2^	0.686	0.761	0.777	0.747	0.761	0.665	0.799
SD	0.048	0.037	0.036	0.038	0.037	0.048	0.034
APRE (%)	0.204	−0.067	−0.087	−0.128	−0.043	1.023	0.052
AAPRE (%)	1.968	2.061	1.891	2.163	2.137	2.130	1.784
RMSE	4.116	3.176	3.061	3.263	3.180	4.261	2.923
Num. of data	287	287	287	287	287	287	287

The validation of the proposed models is supported by consistently low values of RMSE, AAPRE, SD, and APRE across the training, testing, and overall datasets. As shown in Table 3, there are no significant discrepancies between the training and testing sets for any of the developed models, indicating that overfitting is not a pervasive issue.

Among the algorithms, AdaBoost-DT provides satisfactory predictions, but its accuracy is lower than that of the LM-MLP and SCG-MLP models. The statistical metrics further suggest that RB-MLP and BR-MLP are reliable, ranking just after SCG-MLP. In contrast, KNN exhibits the highest prediction errors among the evaluated models.

Specifically, the LM-MLP model achieves the following performance for relative density prediction: R^2^, APRE, SD, AAPRE, and RMSE for the training set are 0.777, − 0.138%, 0.038, 1.927%, and 3.171; for the testing set, they are 0.779, 0.110%, 0.029, 1.745%, and 2.578; and for the total data points, they are 0.777, − 0.087%, 0.036, 1.891%, and 3.061. Based on these results, the models can be ranked in order of predictive accuracy as follows:

LM-MLP ˃ SCG-MLP ˃ RB-MLP ˃ BR-MLP ˃ AdaBoost-DT > KNN.

**Fig. 5:**
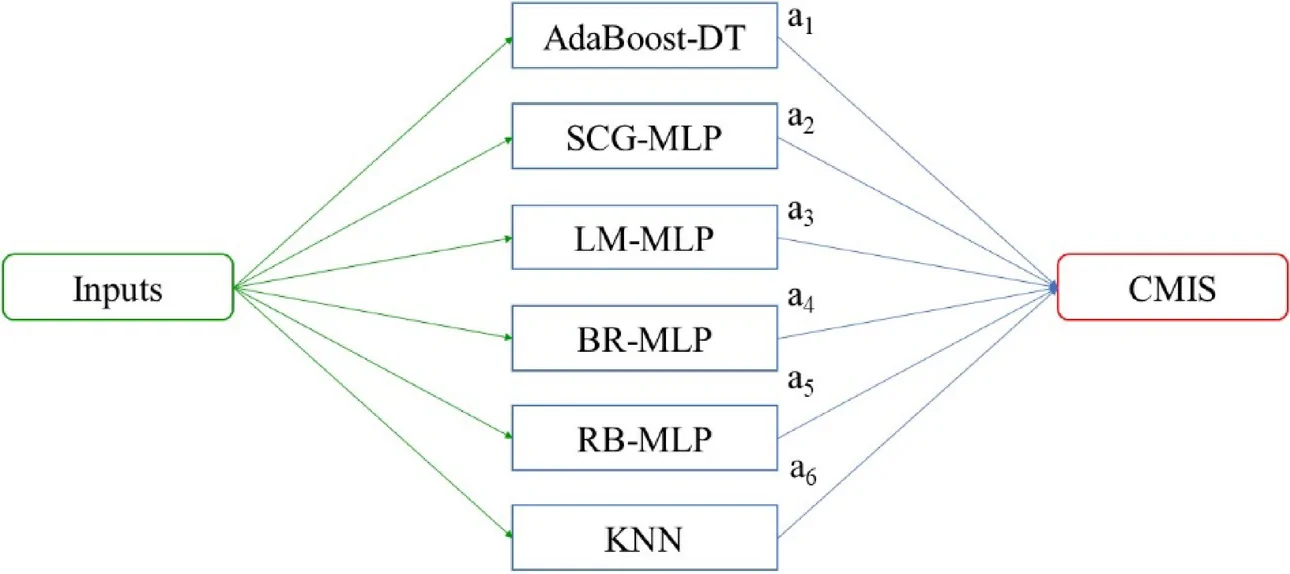
Schematic illustration of the CMIS model in this work.

Finally, a Committee Machine Intelligent System (CMIS) was used to integrate all the individual models. By applying multiple linear regression to determine the optimal coefficients, the CMIS model is expressed by the following equation:

11 $$\begin{aligned} RD_{{CMIS}} = & a_{1} RD_{{AdaBoost - DT}} + a_{2} RD_{{MLP - SCG}} + a_{3} RD_{{MLP - LM}} \\ & + a_{4} RD_{{MLP - BR}} + a_{5} RD_{{MLP - RB}} + a_{6} RD_{{KNN}} \\ \end{aligned}$$

where the values for *a*_1_ through *a*_6_ are:

a_1_ = 0.169322; a_2_ = 0.073555; a_3_ = 0.546554; a_4_ = 0.000033; a_5_ = 0.020769; a_6_ = 0.191011;

Figure 5 presents a schematic diagram of the CMIS model. As indicated in Table 3, the CMIS model demonstrates the highest predictive accuracy, achieving R^2^, APRE, SD, AAPRE, and RMSE values of 0.799, 0.052%, 0.034, 1.784%, and 2.923, respectively, for the overall dataset.

### Visual assessment of the models

**Fig. 6:**
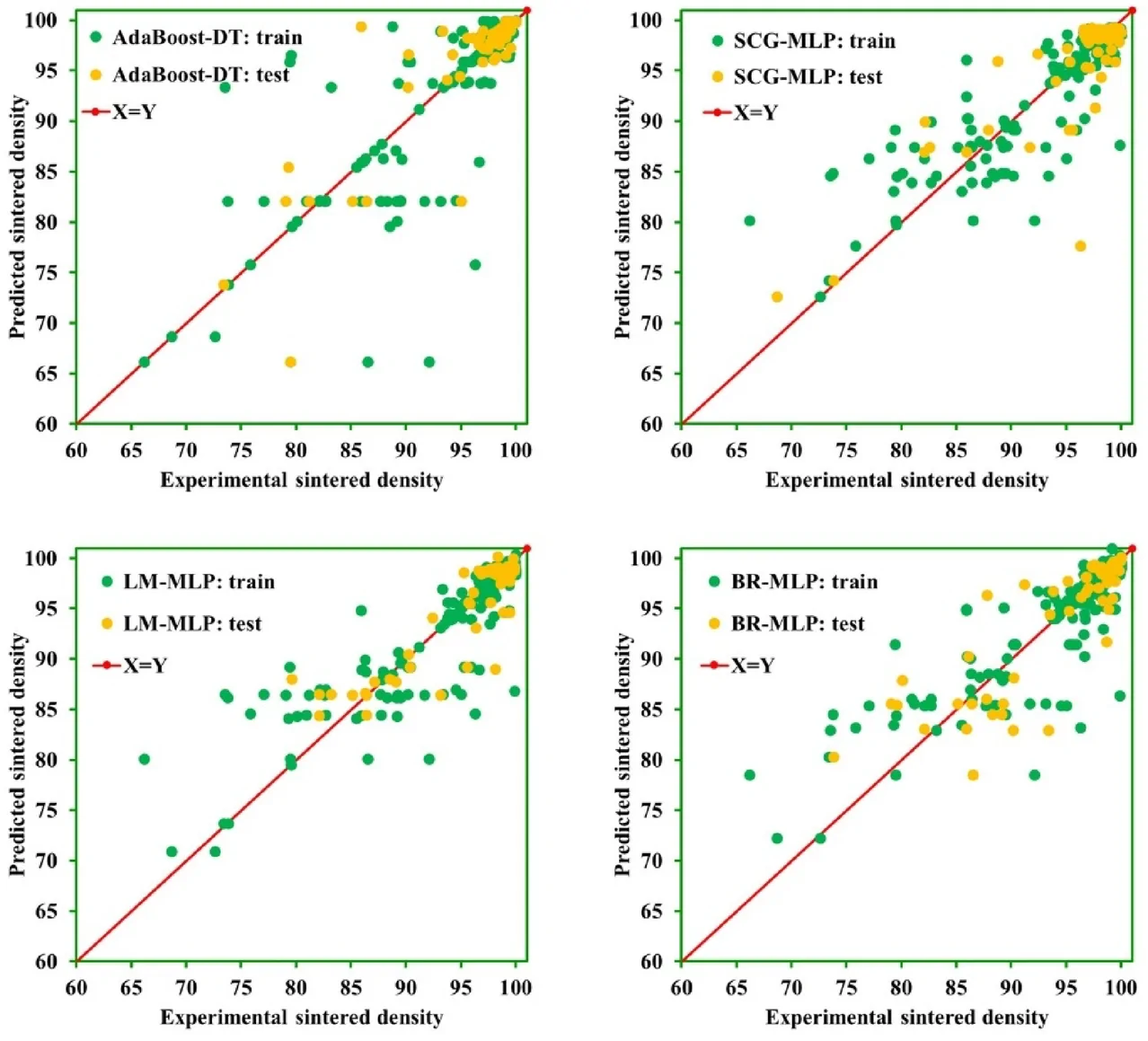

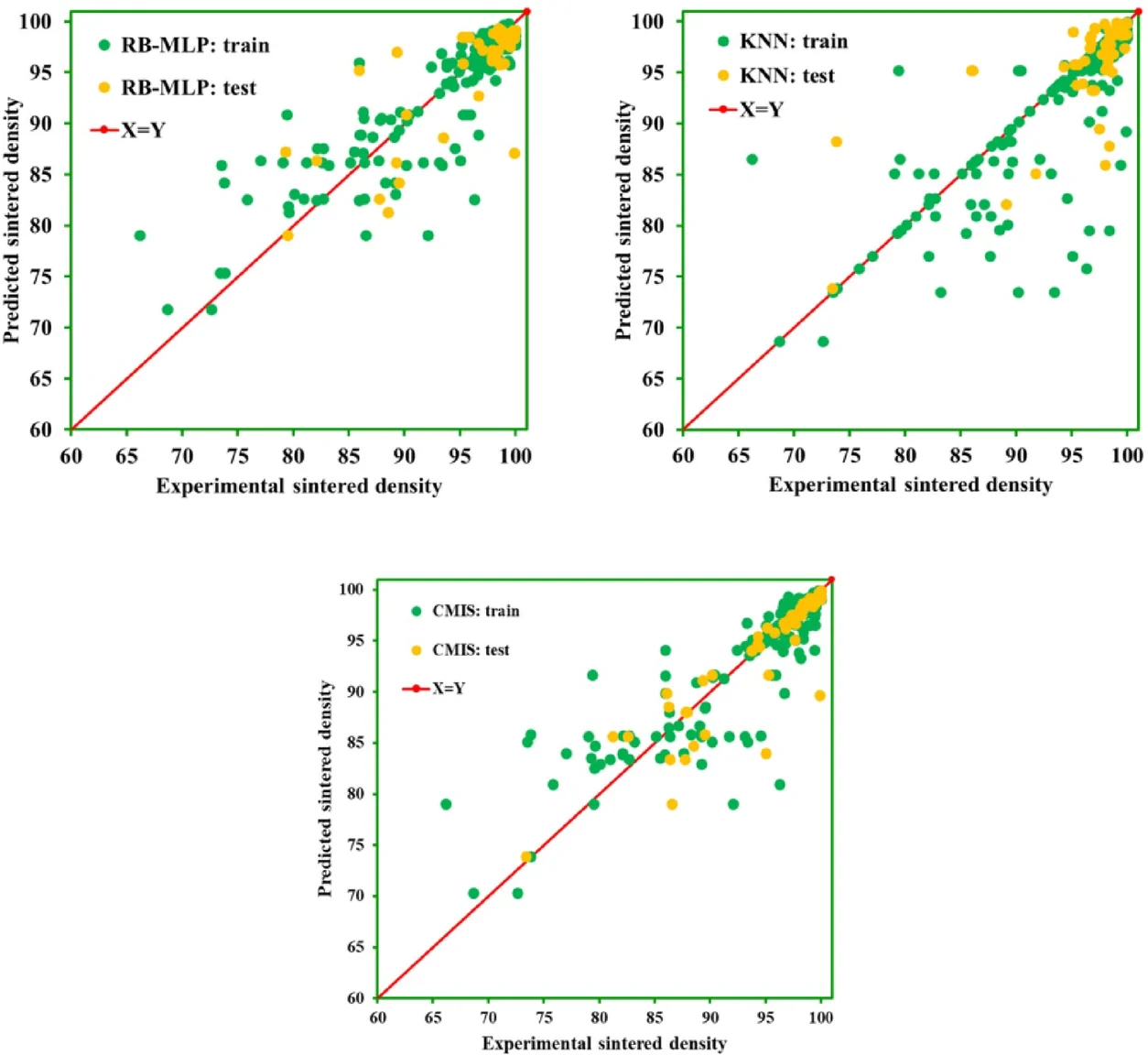
Cross plot showing the correlation between predicted and experimental relative density for the training and testing datasets.

Figure 6 presents cross plots of the predicted relative density versus the corresponding experimental values for both the training and testing sets, providing a visual representation of the prediction errors for the developed models. All models exhibit a strong correlation between predicted and measured relative densities, as evidenced by the close alignment of data points along the 45° line.

For the CMIS model, predictions for both training and testing datasets are tightly clustered around the line of unit slope, indicating minimal dispersion and consistent performance across all data points. Furthermore, the scatter of points does not indicate any systematic underestimation or overestimation, highlighting the reliability and accuracy of the CMIS approach.

**Fig. 7:**
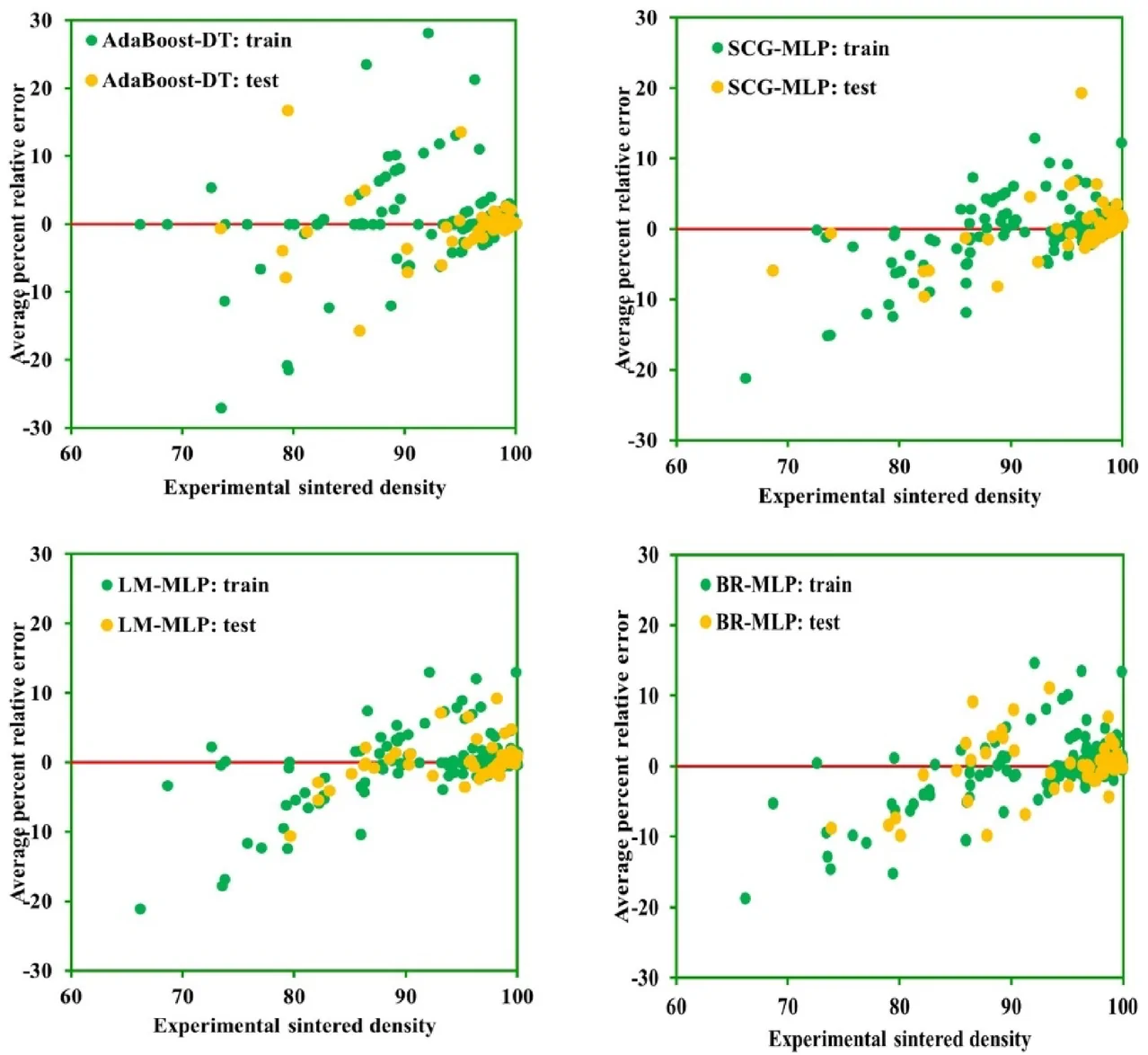

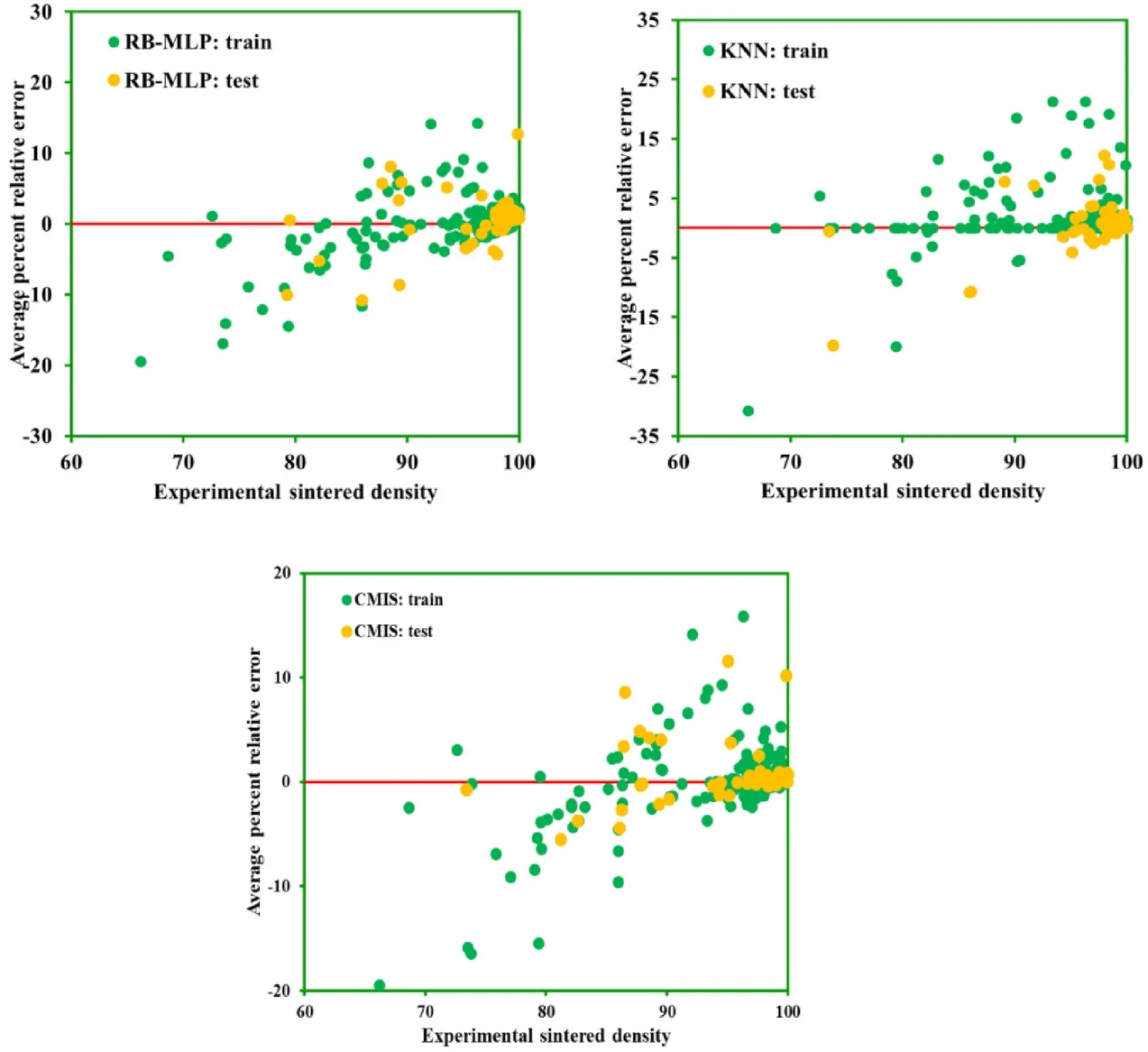
Average percent relative error (APRE) comparing predicted and experimental relative density for both the training and testing datasets.

Figure 7 illustrates the error distribution for the CMIS model and the other models developed in this study for both the training and testing datasets. For the CMIS model, most data points are closely clustered around the zero-error line across the full range of experimental relative density values, with the maximum relative deviation between predicted and observed values reaching − 19.45%. This demonstrates the high consistency and reliability of the CMIS predictions compared to the experimental data. In contrast, the AdaBoost-DT and KNN models exhibit a substantial number of points positioned far from the zero-error line, indicating larger and less consistent prediction errors.

**Fig. 8:**
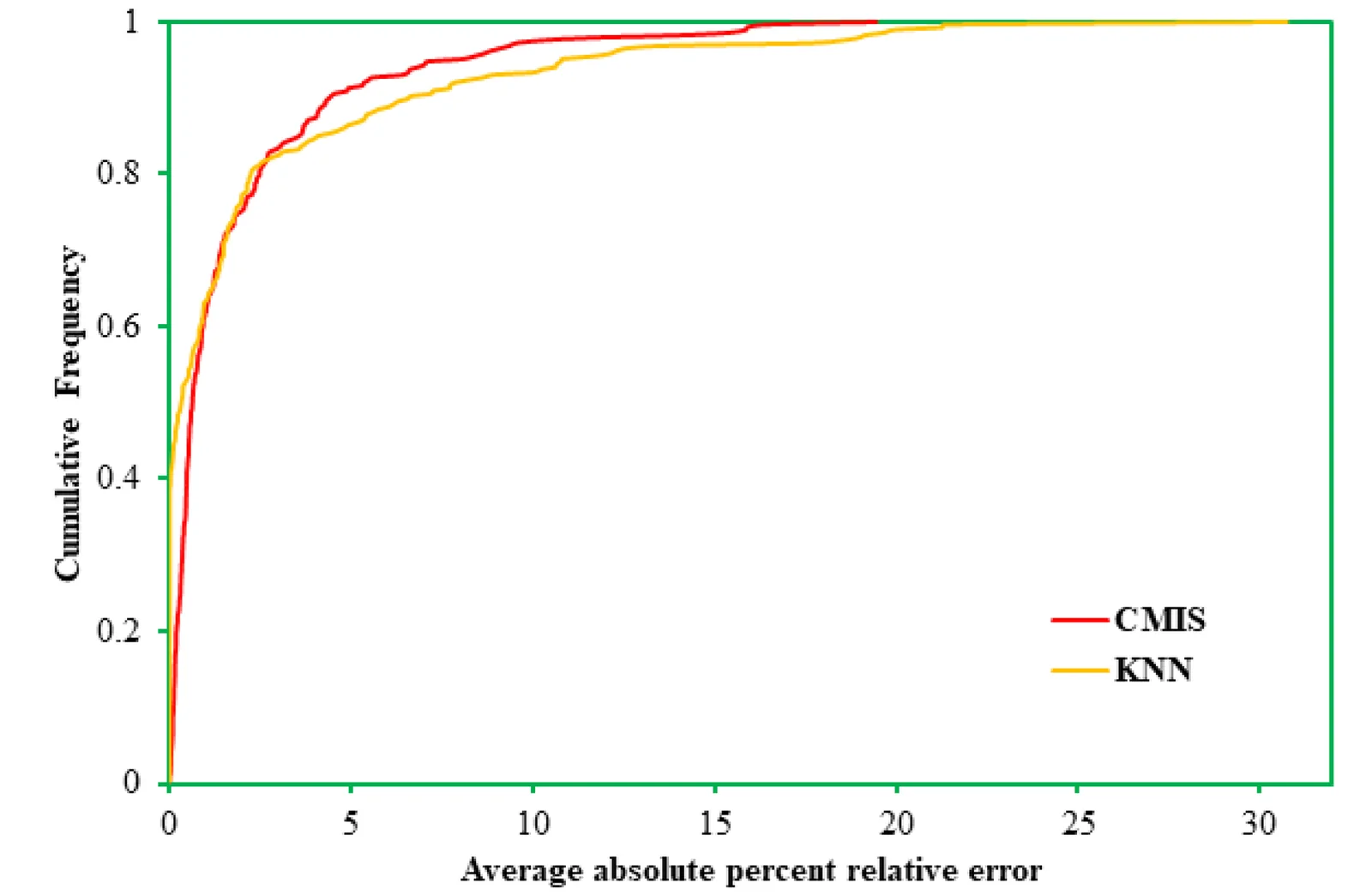
Cumulative frequency curves of prediction errors for the CMIS and KNN models.

Figure 8 shows the cumulative frequency of prediction errors, providing a clear visual comparison between the best and worst-performing models in this study: the CMIS and KNN models. The CMIS model demonstrates the highest cumulative frequency of low prediction errors. Specifically, approximately 90% of the predicted relative density values for the CMIS, LM-MLP, SCG-MLP, RB-MLP, BR-MLP, AdaBoost-DT, and KNN models fall within estimation errors of 4.45%, 5.35%, 5.88%, 5.69%, 6.28%, 6.32%, and 6.64%, respectively. These results highlight the superior accuracy and effectiveness of the CMIS approach for predicting relative density.

### A comparison of the developed models with existing literature models

**Fig. 9:**
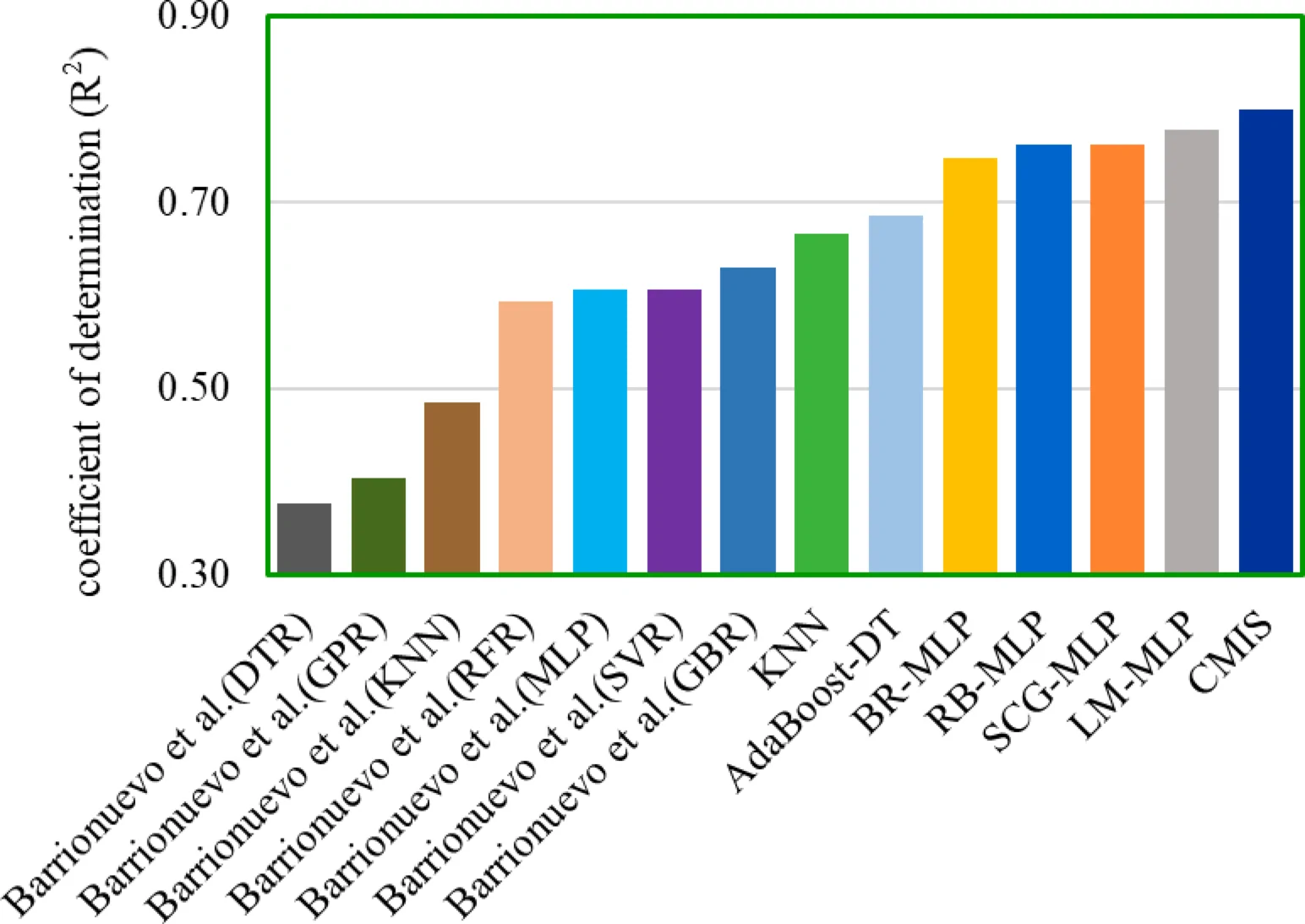
Comparison of the relative density models developed in this study (CMIS, LM-MLP, SCG-MLP, RB-MLP, BR-MLP, AdaBoost-DT, and KNN) with the best-performing model reported in the literature.

Figure 9 compares the coefficient of determination (R^2^) of the models developed in this study with those reported in the literature. A correlation coefficient closer to 1 indicates higher predictive accuracy. The R^2^ values for the machine learning models developed here are as follows: AdaBoost-DT, 0.686; SCG-MLP, 0.761; LM-MLP, 0.777; BR-MLP, 0.747; RB-MLP, 0.761; KNN, 0.665; and CMIS, 0.799. The highest R^2^ value achieved by the CMIS model indicates that it is the most accurate approach among those investigated in this work.

Compared to existing literature^35^, the models developed here demonstrate substantially higher accuracy. For instance, the best-performing model in the referenced study, the gradient boosting regressor (GBR), achieved an R^2^ value of 0.629. This improvement in predictive performance may be attributed to the specific model design and the advanced algorithms employed in this study.

### Sensitivity analysis of the CMIS model

Sensitivity analysis evaluates how variations in a model’s input parameters affect its output. A robust approach for quantifying this relationship is relevance factor analysis, which measures the influence of each input on the output. In this study, sensitivity analysis was conducted for the proposed CMIS model. The relevancy factor (*r*) indicates the magnitude of each input’s effect on the output, with higher absolute values reflecting a stronger influence^25^.

**Fig. 10:**
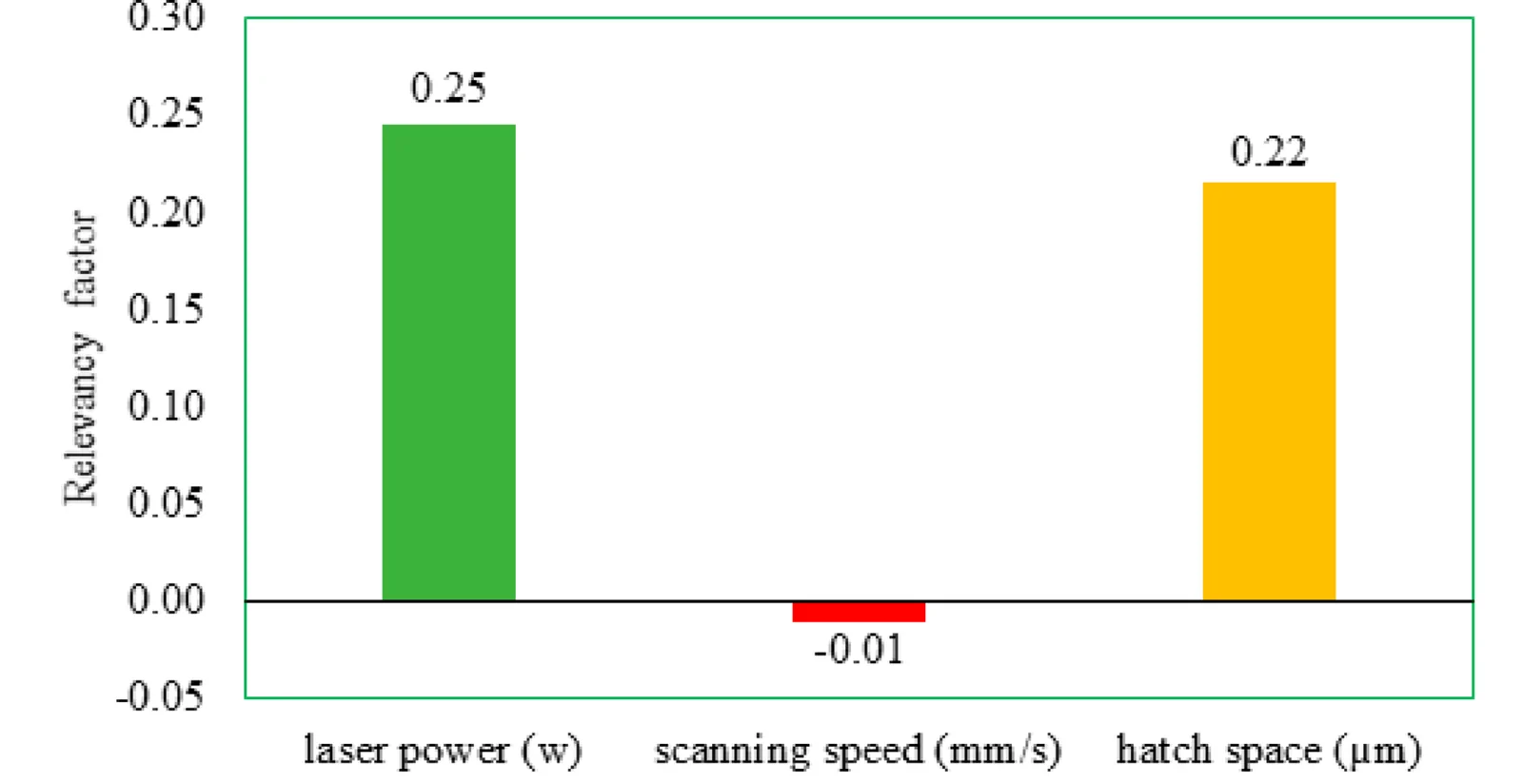
Sensitivity analysis of the developed CMIS model showing the influence of input parameters on relative density.

Figure 10 shows the impact of different input parameters on the relative sintered density. The analysis reveals that relative density is most strongly affected by laser power and hatch spacing. Scanning speed has the least influence, exerting a negative effect; increasing the scanning speed tends to decrease the relative density. In contrast, increasing laser power or hatch spacing positively affects the relative density, leading to higher densification values. Although the sensitivity analysis indicates a relatively weaker influence of scanning speed compared to other parameters, this observation should be interpreted with caution. In L‑PBF processes, scanning speed plays a critical role in melt pool dynamics and defect formation. The reduced sensitivity observed in this study may be associated with the limited parameter ranges available in the compiled dataset or correlations between the input variables within the literature data.

### Applicability domain and outlier detection of the proposed CMIS model

A primary purpose of outlier detection techniques is to identify data points that deviate significantly from the rest of the dataset. One effective approach for detecting such points is the leverage method. This method employs the Hat matrix, which incorporates both the predicted and actual values from the model, along with the standardised residuals.

The Hat indices are calculated using the following formula, based on the Hat matrix (H):

12 $${\text{H }} = X{\text{ }}({\text{ }}X^{t} ~X)^{{ - 1}} X^{t}$$

In this equation, *X* represents an (n × m) matrix where *m* corresponds to the number of input parameters and n is the number of data points, and t denotes the transpose of the matrix.

To visually assess outliers, a William’s plot is generated using the computed Hat matrix (H). The warning leverage (H*) is calculated as H* = $\:\frac{3\:(\:x\:+\:1\:)}{n}$, where x is the number of input parameters, and n is the total number of data points. For the model and its predictions to be considered statistically valid and reliable, the majority of data points should fall within the ranges of 0 ≤ H_*ii*_ ≤ H* for leverage values and − 3 ≤ *R* ≤ 3 for the standardised residuals R ^52^.

**Fig. 11:**
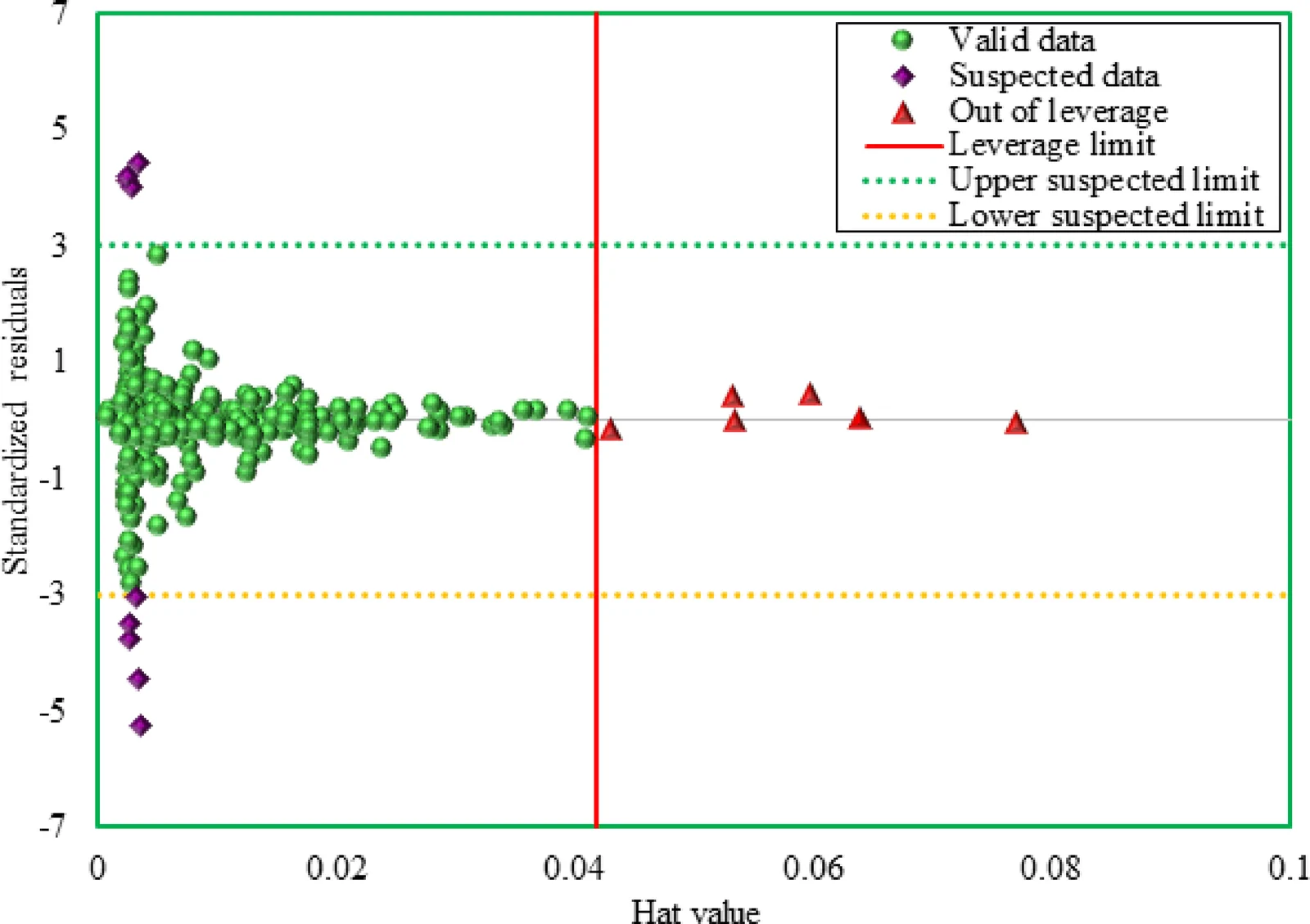
The CMIS model’s developed Williams plot.

As shown in Fig. 11, the leverage analysis indicates that most data points lie within the valid range. However, approximately 5% of the data, corresponding to fifteen points, fall outside the model’s applicability domain. This outcome confirms the statistical validity of the model and supports the accuracy of the experimental data.

The observed predictive trends can be physically interpreted in the context of melt pool dynamics and defect formation mechanisms in L-PBF. Increasing laser power enhances energy input, promotes stable melt pool formation, reduces melt viscosity, and facilitates liquid flow into pores, thereby increasing relative density. Hatch spacing plays a dual role: excessively large spacings result in insufficient track overlap and the formation of lack-of-fusion defects, while overly small spacings cause localised energy accumulation and melt pool instability, both of which negatively impact densification. Scanning speed exhibits an inverse relationship with relative density because higher speeds reduce laser–material interaction time, leading to incomplete melting.

The superior performance of the CMIS model can be attributed to its ability to integrate complementary learning mechanisms that capture these complex physical interactions. While individual models tend to identify specific nonlinear patterns associated with particular process conditions, the CMIS framework effectively combines their strengths, enabling simultaneous representation of multiple densification mechanisms. This multi-model integration reflects the inherently multi-physical nature of the L-PBF process and explains the enhanced accuracy, robustness, and generalisation capability observed in the CMIS predictions.

In this study, data points located outside the defined applicability domain are not considered erroneous; rather, they indicate conditions under which model predictions may be less reliable. This approach ensures that performance metrics are not artificially inflated by extrapolation and provides a transparent means of identifying influential or outlying observations.

Although the dataset is finite, it covers a broad and representative range of L-PBF processing conditions reported for 316 L stainless steel. Incorporating data from multiple independent studies introduces variability that enhances model generalisation while also defining clear applicability limits. Consequently, the proposed framework is intended for interpolation within the validated process window, as supported by uncertainty and applicability domain analyses, rather than for extrapolation beyond experimentally validated conditions.

## Conclusions

To evaluate the relative density of 316 L stainless steel, seven predictive models were developed in this study using data compiled from various literature sources. A total of 287 experimental data points were collected, considering key L-PBF process parameters: laser power, scanning speed, and hatch spacing. Based on the results, the following conclusions can be drawn:

1. The models developed in this study can accurately predict relative density, with the CMIS model demonstrating the highest reliability.
2. The CMIS model achieves excellent predictive performance, with AAPRE values of 1.836% for the training set, 1.575% for the testing set, and 1.784% for the overall dataset.
3. The predictive accuracy of the models developed in this study can be ranked as follows:
  CMIS > LM-MLP > SCG-MLP > RB-MLP > BR-MLP > AdaBoost-DT > KNN.
4. A comparison between the newly developed soft computing models and previously reported methods demonstrated that the proposed CMIS model outperforms all earlier approaches in terms of predictive accuracy.
5. Leverage analysis showed that the vast majority of data points fall within the valid domain, with only a small fraction (approximately 5% of the dataset) lying outside the defined leverage limits, indicating potential outliers.
6. Sensitivity analysis revealed that laser power is one of the most influential input parameters affecting relative density, while hatch spacing requires careful optimisation: excessively wide spacing leads to poor track overlap and lack-of-fusion porosity, whereas excessively narrow spacing causes localised energy accumulation and melt pool instability, both of which reduce densification.

## Data Availability

The datasets used during the current study are available from the corresponding author upon reasonable request.

## References

[1] Armstrong, M., Mehrabi, H. & Naveed, N. An overview of modern metal additive manufacturing technology. *J. Manuf. Process.* **84**, 1001–1029 (2022).

[2] Li, Y., Su, C. & Zhu, J. Comprehensive review of wire arc additive manufacturing: Hardware system, physical process, monitoring, property characterization, application and future prospects. *Results Eng.* **13**, 100330 (2022).

[3] Liu, Z. et al. Additive manufacturing of metals: Microstructure evolution and multistage control. *J. Mater. Sci. Technol.* **100**, 224–236 (2022).

[4] Lu, B. & Li, D. Development of the additive manufacturing (3D printing) technology. *Mach. Building Autom.* **42**, 1–4 (2013).

[5] Mercurio, V., Calignano, F. & Iuliano, L. Sustainable production of AlSi10Mg parts by laser powder bed fusion process. *Int. J. Adv. Manuf. Technol.* **125**, 3117–3133 (2023).

[6] Colosimo, B. M., Huang, Q., Dasgupta, T. & Tsung, F. Opportunities and challenges of quality engineering for additive manufacturing. *J. Qual. Technol.* **50**, 233–252 (2018).

[7] Guo, S. et al. Machine learning for metal additive manufacturing: Towards a physics-informed data-driven paradigm. *J. Manuf. Syst.* **62**, 145–163 (2022).

[8] Riveiro, A. et al. Laser additive manufacturing processes for near net shape components. *Near Net Shape Manuf. Proc.*. **105**, 141 (2019).

[9] Calignano, F., Cattano, G. & Manfredi, D. Manufacturing of thin wall structures in AlSi10Mg alloy by laser powder bed fusion through process parameters. *J. Mater. Process. Technol.* **255**, 773–783 (2018).

[10] Pfaff, A. et al. Resource analysis model and validation for selective laser melting, constituting the potential of lightweight design for material efficiency. *Sustain. Prod. Consum.* **21**, 182–191 (2020).

[11] Pandiyan, V. et al. Deep learning-based monitoring of laser powder bed fusion process on variable time-scales using heterogeneous sensing and operando X-ray radiography guidance. *Additive Manuf.* **58**, 103007 (2022).

[12] Pandiyan, V., Wróbel, R., Leinenbach, C. & Shevchik, S. Optimizing in-situ monitoring for laser powder bed fusion process: Deciphering acoustic emission and sensor sensitivity with explainable machine learning. *J. Mater. Process. Technol.* **321**, 118144 (2023).

[13] DebRoy, T. et al. Additive manufacturing of metallic components–process, structure and properties. *Prog. Mater. Sci.* **92**, 112–224 (2018).

[14] Bidare, P., Bitharas, I., Ward, R., Attallah, M. & Moore, A. J. Fluid and particle dynamics in laser powder bed fusion. *Acta Mater.* **142**, 107–120 (2018).

[15] Ly, S., Rubenchik, A. M., Khairallah, S. A., Guss, G. & Matthews, M. J. Metal vapor micro-jet controls material redistribution in laser powder bed fusion additive manufacturing. *Sci. Rep.* **7**, 4085 (2017). 28642468 10.1038/s41598-017-04237-z PMC5481335

[16] Tan, X. et al. Graded microstructure and mechanical properties of additive manufactured Ti–6Al–4V via electron beam melting. *Acta Mater.* **97**, 1–16 (2015).

[17] Thijs, L., Verhaeghe, F., Craeghs, T., Van Humbeeck, J. & Kruth J.-P. A study of the microstructural evolution during selective laser melting of Ti–6Al–4V. *Acta Mater.* **58**, 3303–3312 (2010).

[18] Scipioni Bertoli, U., Wolfer, A. J., Matthews, M. J., Delplanque, J. P. & Schoenung, J. M. On the limitations of volumetric energy density as a design parameter for selective laser melting. *Mater. Design*. **113**, 331–340. 10.1016/j.matdes.2016.10.037 (2017).

[19] Khairallah, S. A., Anderson, A. T., Rubenchik, A. & King, W. E. Laser powder-bed fusion additive manufacturing: Physics of complex melt flow and formation mechanisms of pores, spatter, and denudation zones. *Acta Mater.* **108**, 36–45. 10.1016/j.actamat.2016.02.014 (2016).

[20] Dilip, J. J. S. et al. Influence of processing parameters on the evolution of melt pool, porosity, and microstructures in Ti-6Al-4V alloy parts fabricated by selective laser melting. *Prog. Additive Manuf.* **2**, 157–167. 10.1007/s40964-017-0030-2 (2017).

[21] Liu, Q. et al. Machine-learning assisted laser powder bed fusion process optimization for AlSi10Mg: New microstructure description indices and fracture mechanisms. *Acta Mater.* **201**, 316–328 (2020).

[22] Zhang, Y., Yang, S., Dong, G. & Zhao, Y. F. Predictive manufacturability assessment system for laser powder bed fusion based on a hybrid machine learning model. *Additive Manuf.* **41**, 101946 (2021).

[23] Mythreyi, O., Srinivaas, M. R., Kumar, A. & Jayaganthan, R. T. Machine-learning-based prediction of corrosion behavior in additively manufactured Inconel 718. *Data* 6, 80 (2021).

[24] Sanchez, S., Rengasamy, D., Hyde, C. J., Figueredo, G. P. & Rothwell, B. Machine learning to determine the main factors affecting creep rates in laser powder bed fusion. *J. Intell. Manuf.* **32**, 2353–2373 (2021). 34720456 10.1007/s10845-021-01785-0 PMC8550259

[25] Asnaashari, S., Shateri, M., Hemmati-Sarapardeh, A. & Band, S. S. Modeling of the sintered density in Cu-Al alloy using machine learning approaches. *ACS Omega*. **8**, 28036–28051 (2023). 37576653 10.1021/acsomega.2c07278 PMC10413372

[26] Hassanin, H., Alkendi, Y., Elsayed, M., Essa, K. & Zweiri, Y. Controlling the properties of additively manufactured cellular structures using machine learning approaches. *Adv. Eng. Mater.* **22**, 1901338 (2020).

[27] Maitra, V., Shi, J. & Lu, C. Robust prediction and validation of as-built density of Ti-6Al-4V parts manufactured via selective laser melting using a machine learning approach. *J. Manuf. Process.* **78**, 183–201. 10.1016/j.jmapro.2022.04.020 (2022).

[28] Scime, L. & Beuth, J. Anomaly detection and classification in a laser powder bed additive manufacturing process using a trained computer vision algorithm. *Additive Manuf.* **19**, 114–126 (2018).

[29] Gobert, C., Reutzel, E. W., Petrich, J., Nassar, A. R. & Phoha, S. Application of supervised machine learning for defect detection during metallic powder bed fusion additive manufacturing using high resolution imaging. *Additive Manuf.* **21**, 517–528 (2018).

[30] Alaña, M., Cutolo, A., Ruiz de Galarreta, S. & Van Hooreweder, B. Influence of relative density on quasi-static and fatigue failure of lattice structures in Ti6Al4V produced by laser powder bed fusion. *Sci. Rep.* **11**, 19314 (2021). 34588524 10.1038/s41598-021-98631-3 PMC8481248

[31] Wang, D., Liu, Y., Yang, Y. & Xiao, D. Theoretical and experimental study on surface roughness of 316L stainless steel metal parts obtained through selective laser melting. *Rapid Prototyp. J.* **22**, 706–716 (2016).

[32] Jiang, H. Z. et al. Effect of process parameters on defects, melt pool shape, microstructure, and tensile behavior of 316L stainless steel produced by selective laser melting. *Acta Metall. Sinica (Engl. Lett.)*. **34**, 495–510 (2021).

[33] Sun, J. et al. Study of microstructure and properties of 316L with selective laser melting based on multivariate interaction influence. *Adv. Mater. Sci. Eng.* 8404052 (2020). (2020).

[34] Deng, Y., Mao, Z., Yang, N., Niu, X. & Lu, X. Collaborative optimization of density and surface roughness of 316L stainless steel in selective laser melting. *Materials* **13**, 1601 (2020). 32244593 10.3390/ma13071601 PMC7178480

[35] Barrionuevo, G. O., Ramos-Grez, J. A., Walczak, M. & Betancourt, C. A. Comparative evaluation of supervised machine learning algorithms in the prediction of the relative density of 316L stainless steel fabricated by selective laser melting. *Int. J. Adv. Manuf. Technol.* **113**, 419–433 (2021).

[36] Laleh, M. et al. Two and three-dimensional characterisation of localised corrosion affected by lack-of-fusion pores in 316L stainless steel produced by selective laser melting. *Corros. Sci.* **165**, 108394 (2020).

[37] Li, B., Wang, T., Li, P., Wang, S. & Wang, L. Selective laser melting of 316L stainless steel: Influence of Co-Cr-Mo-W addition on corrosion resistance. *Metals* **11**, 597 (2021).

[38] Diaz Vallejo, N. et al. Process optimization and microstructure analysis to understand laser powder bed fusion of 316L stainless steel. *Metals* **11**, 832 (2021).

[39] Simchi, A. Effect of C and Cu addition on the densification and microstructure of iron powder in direct laser sintering process. *Mater. Lett.* **62**, 2840–2843 (2008).

[40] Guo, C. et al. Effect of processing parameters on surface roughness, porosity and cracking of as-built IN738LC parts fabricated by laser powder bed fusion. *J. Mater. Process. Technol.* **285**, 116788 (2020).

[41] Altman, N. S. An introduction to kernel and nearest-neighbor nonparametric regression. *Am. Stat.* **46**, 175–185 (1992).

[42] Thanh Noi, P. & Kappas, M. Comparison of random forest, k-nearest neighbor, and support vector machine classifiers for land cover classification using Sentinel-2 imagery. *Sensors* **18**, 18 (2017). 29271909 10.3390/s18010018 PMC5796274

[43] Abdolrasol, M. G. et al. Artificial neural networks based optimization techniques: A review. *Electronics* **10**, 2689 (2021).

[44] Liu, X., Tian, S., Tao, F. & Yu, W. A review of artificial neural networks in the constitutive modeling of composite materials. *Compos. Part. B: Eng.* **224**, 109152 (2021).

[45] La Fé-Perdomo, I., Ramos-Grez, J., Mujica, R. & Rivas, M. Surface roughness Ra prediction in Selective Laser Melting of 316L stainless steel by means of artificial intelligence inference. *J. King Saud Univ. Eng. Sci.* **35**, 148–156 (2023).

[46] Cheng, J., Ling, Y., De Waele, W. & An, A. N. N. Hardness prediction tool based on a finite element implementation of a thermal–metallurgical model for mild steel produced by WAAM. *Metals* **14**, 556 (2024).

[47] Parvizi, S., Hafizpour, H., Sadrnezhaad, S. & Akhondzadeh, A. Abbasi Gharacheh, M. Neural network prediction of mechanical properties of porous NiTi shape memory alloy. *Powder Metall.* **54**, 450–454 (2011).

[48] Zhao, Q. et al. in *Proceedings of the 24th International Conference on Hybrid Systems: Computation and Control.* 1–11.

[49] Hoffmann, L. F. S., Bizarria, F. C. P. & Bizarria, J. W. P. Detection of liner surface defects in solid rocket motors using multilayer perceptron neural networks. *Polym. Test.* **88**, 106559 (2020).

[50] Rasamoelina, A. D., Adjailia, F. & Sinčák, P. in *2020 IEEE 18th World Symposium on Applied Machine Intelligence and Informatics (SAMI).* 281–286 (IEEE).

[51] Pasang, S. et al. Analysis of road accidents due to obstruction in sight distance caused by foggy weather using Geographic Information System (GIS) and Artificial Neural Network (ANN) at Jumja along Thimphu-Phuentsholing Highway (Asian Highway 48).

[52] Hemmati-Sarapardeh, A., Varamesh, A., Husein, M. M. & Karan, K. On the evaluation of the viscosity of nanofluid systems: Modeling and data assessment. *Renew. Sustain. Energy Rev.* **81**, 313–329 (2018).

[53] Moré, J. J. in *Numerical analysis: proceedings of the biennial Conference held at Dundee, June 28–July 1, 1977.* 105–116 (Springer).

[54] Ranganathan, A. The levenberg-marquardt algorithm. *Tutoral LM algorithm*. **11**, 101–110 (2004).

[55] Yu, H. & Wilamowski, B. M. in *Intell. Syst.* 12-11-12-16CRC Press, (2018).

[56] Fun, M. H. & Hagan, M. T. in *Proceedings of International Conference on Neural Networks (ICNN’96).* 468–473 (IEEE).

[57] Yue, Z., Songzheng, Z. & Tianshi, L. in *International Conference on Business Management and Electronic Information.* 483–487 (IEEE). 483–487 (IEEE). (2011).

[58] Ameli, F., Hemmati-Sarapardeh, A., Schaffie, M., Husein, M. M. & Shamshirband, S. Modeling interfacial tension in N2/n-alkane systems using corresponding state theory: Application to gas injection processes. *Fuel* **222**, 779–791 (2018).

[59] Schapire, R. E. The strength of weak learnability. *Mach. Learn.* **5**, 197–227 (1990).

[60] Freund, Y. & Schapire, R. E. A decision-theoretic generalization of on-line learning and an application to boosting. *J. Comput. Syst. Sci.* **55**, 119–139 (1997).

[61] Loh, W. Y. Fifty years of classification and regression trees. *Int. Stat. Rev.* **82**, 329–348 (2014). 10.1111/insr.12060 PMC4380222 25844011

[62] Song, Y. Y. & Ying, L. Decision tree methods: applications for classification and prediction. *Shanghai Archives Psychiatry*. **27**, 130 (2015). 10.11919/j.issn.1002-0829.215044 PMC4466856 26120265

[63] Patel, N. & Upadhyay, S. Study of various decision tree pruning methods with their empirical comparison in WEKA. *Int. J. Comput. Appl.* **60** (2012).

[64] Nilsson, N. J. Learning machines. (1965).

[65] Haykin, S. & Network, N. A comprehensive foundation. *Neural Netw.* **2**, 41 (2004).

[66] Perrone, M. P. & Cooper, L. N. in *How we learn; How we remember: Toward an understanding of brain and neural systems: Selected papers of Leon N Cooper* 342–358World Scientific, (1995).

[67] Hashem, S. & Schmeiser, B. *Approximating a function and its derivatives using MSE-optimal linear combinations of trained feedforward neural networks*Citeseer,. (1993).

[68] Shokrollahi, A., Tatar, A. & Safari, H. On accurate determination of PVT properties in crude oil systems: Committee machine intelligent system modeling approach. *J. Taiwan Inst. Chem. Eng.* **55**, 17–26 (2015).

